# How tumors escape the immune system

**DOI:** 10.7554/eLife.97881

**Published:** 2024-05-14

**Authors:** Stefanie Schmieder

**Affiliations:** 1 Boston Children's Hospital, Harvard Medical School Boston United States

**Keywords:** exosomes, hepatocellular carcinoma, liver cancer, immunotherapy, Beta-catenin

## Abstract

Mutations in the gene for β-catenin cause liver cancer cells to release fewer exosomes, which reduces the number of immune cells infiltrating the tumor.

**Related research article** Dantzer C, Vaché J, Brunel A, Mahouche I, Raymond AA, Dupuy JW, Petrel M, Bioulac-Sage P, Perrais D, Dugot-Senant N, Verdier M, Bessette B, Billottet C, Moreau V. 2024. Emerging role of oncogenic ß-catenin in exosome biogenesis as a driver of immune escape in hepatocellular carcinoma. *eLife* **13**:RP95191. doi: 10.7554/eLife.95191.

All cells in the body release small nano-sized vesicles known as exosomes into the space around them. These vesicles contain a diverse array of biological materials, such as nucleic acids, proteins, lipids and other metabolites that represent the ‘parent cell’ they came from (Figure 1A).

**Figure 1.**
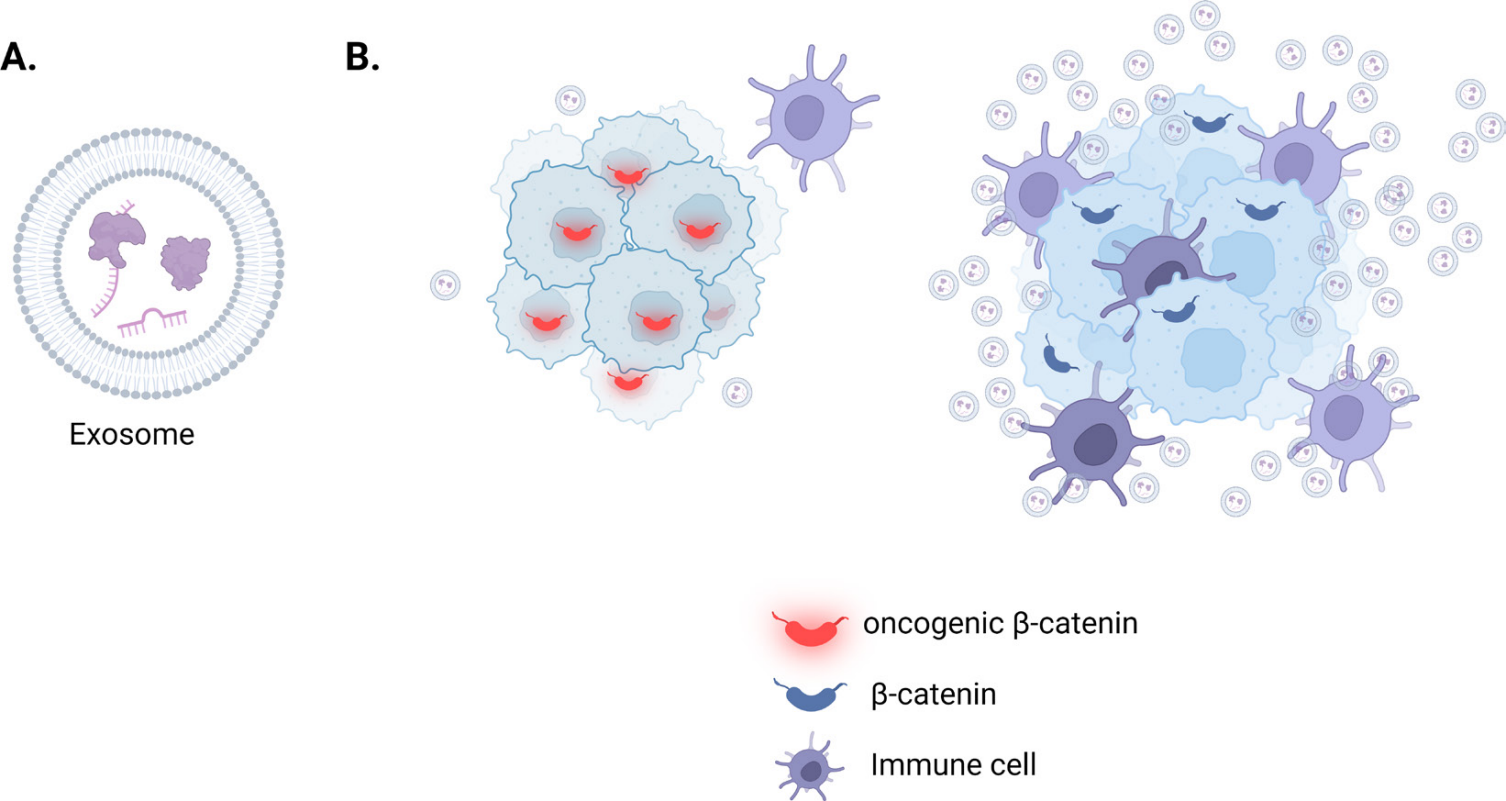
The role of exosomes in liver cancers associated with oncogenic β-catenin. (**A**) Exosomes are small, membrane-encapsulated vesicles that cells release into their environment. The content of each vesicle is reflective of the cell it came from and may include nucleic acids, proteins, metabolites and lipids (purple). (**B**) Hepatocellular carcinoma cells (light blue with dark blue outline), which carry a mutated version of β-catenin (also known as oncogenic β-catenin; red), secrete fewer exosomes due to downregulating the expression of genes required to build and release these vesicles (left). The lack of exosomes in the tumor microenvironment reduces the number of immune cells (purple) infiltrating these tumors compared to other types of liver cancer which express a non-mutated version of β-catenin (dark blue; right).

When exosomes were first discovered in 1981, they were thought to just be another mechanism cells use to relieve themselves of unwanted materials (Trams et al., 1981; Johnstone et al., 1987; Harding et al., 1983; Kucharzewska et al., 2013). However, more recently exosomes have emerged as important players in how cells communicate with each other, even when they are far apart. Exosomes released from the parent cell are taken up by or fused into the membrane of a recipient cell. This delivery of active biological material can influence the behavior of the recipient cell, such as the genes it expresses and how it responds to stimuli (Valadi et al., 2007; Chen et al., 2014).

In cancer, for example, exosomes released from tumor cells have been shown to promote drug resistance and the local formation of blood vessels (Hu et al., 2015; Chen et al., 2014; Qu et al., 2016). They can also mediate how cancer cells interact with the immune system, such as impairing how immune cells detect and target cancerous cells (Liu et al., 2020; Kalluri and LeBleu, 2020; Ning et al., 2018; Mathieu et al., 2019). Despite these advances, however, much remains unknown about the role exosomes play in cancer and, importantly, what regulates exosome secretion in the parent cell. Now, in eLife, Clotilde Billottet, Violaine Moreau and co-workers – including Camille Dantzer as first author – report how mutations in the gene for β-catenin affect how liver cancer cells secrete exosomes and, in turn, the recruitment of immune cells to the tumor (Dantzer et al., 2024).

Many tumors carry mutations in the gene for β-catenin, including around 30–40% of tumors associated with a type of liver cancer called hepatocellular carcinoma (de L Coste et al., 1998; Rebouissou et al., 2016). This group of tumors has an important unifying feature: they cannot be infiltrated by cells of the immune system, and are therefore resistant to immunotherapy. But it was unclear what was causing this effect.

To investigate, Dantzer et al. (who are based at the University of Bordeaux and University of Limoges) studied a cell-based model of hepatocellular carcinoma in which the mutant β-catenin (also known as oncogenic β-catenin) had been silenced. They found that this increased the expression of genes involved in generating and secreting exosomes (Figure 1B). It also restored the number of exosomes secreted from the cancer cells, suggesting that oncogenic β-catenin represses the secretion of these vesicles.

To better understand the molecular mechanism at play, Dantzer et al. focused their study on two genes that are downregulated when oncogenic β-catenin is present: one of these genes codes for the small GTPase Rab27a, and the other codes for the receptor protein syndecan-4. Further experiments showed that these two genes are repressed in multiple cell models of liver cancer carrying oncogenic β-catenin. This correlation was also observed in tissue samples from patients with liver cancer, where the presence of oncogenic β-catenin corresponded with fewer transcripts of both Rab27a and syndecan-4.

Dantzer et al. also conducted experiments in a cell model of liver cancer in which β-catenin is not mutated. They found that activating the β-catenin pathway decreased the expression of the genes for Rab27a and syndecan-4, thus resulting in reduced exosome secretion. This suggests that β-catenin may be a master regulator of exosome machinery.

Finally, Dantzer et al. investigated the functional consequence of secreting fewer exosomes. To do this, they studied three dimensional models of hepatocellular carcinoma in which the gene for Rab27a had been depleted. The concomitant loss of exosomes resulted in a marked decrease in immune cells infiltrating the tumor models.

While it is still unclear how exosomes derived from liver cancer cells affect the immune system, this elegant study provides important mechanistic information about why immune cells struggle to infiltrate these tumors. However, there are many questions that remain to be answered. For instance, it is unclear if the exosomes secreted from cancer cells carrying oncogenic β-catenin contain the same materials as those secreted from cancer cells containing the non-mutated version of the gene. It is possible that the exosomes released from the cancer cells are transporting tumor-associated antigens that are required to stimulate an immune response. Perhaps most importantly it remains to be seen how general this mechanism of exosome regulation is, and whether β-catenin regulates exosome secretion in other contexts as well. Answering these questions may provide new treatment avenues for diseases in which exosome secretion has been blocked, or is harming tissues and cells.
